# Exosome Therapy: A Novel Approach for Enhancing Estrogen Levels in Perimenopause

**DOI:** 10.3390/ijms25137075

**Published:** 2024-06-27

**Authors:** Samar Alkhrait, Mervat M. Omran, Mohammad Mousaei Ghasroldasht, Hang-Soo Park, Riham Katkhuda, Ayman Al-Hendy

**Affiliations:** 1 Department of Obstetrics and Gynecology, University of Chicago, Chicago, IL 60637, USA; mervatomran@bsd.uchicago.edu (M.M.O.); mmghasroldasht@bsd.uchicago.edu (M.M.G.); 2 Department of Obstetrics and Gynecology, Maternity Hospital-Damascus University, Damascus 011, Syria; 3 Cancer Biology Department, National Cancer Institute—Cairo University, Cairo 11769, Egypt; 4 Department of Biomedical Science, Sunchon National University, Suncheon-si 57922, Republic of Korea; taos0602@gmail.com; 5 Department of Pathology, University of Chicago, Chicago, IL 60637, USA; riham.katkhuda@uchicagomedicine.org

**Keywords:** perimenopause, exosome, estrogen

## Abstract

Perimenopause significantly impacts women’s health globally, often managed with hormone replacement therapy (HRT) despite the associated risks. This study explores a novel alternative exosome therapy, aimed at stimulating estrogen production in ovarian tissues, thus offering a potential non-hormonal treatment for perimenopausal symptoms. Employing ex vivo methodologies, ovarian cortex specimens from perimenopausal women were treated with exosomes derived from human umbilical cord mesenchymal stem cells and cultured under specific conditions (patent number: PCT/US2022/073467). The exosomes were produced under cyclic guanosine monophosphate (cGMP) conditions, ensuring high safety standards. Estrogen levels were quantified using enzyme-linked immunosorbent assay (ELISA), and gene expression changes in estrogen and follicle-stimulating hormone (FSH) receptors were assessed via quantitative polymerase chain reaction (PCR). Immunohistochemistry (IHC) was utilized to evaluate cellular proliferation and apoptotic markers. The results indicated a significant increase in estrogen levels and estrogen receptor-alpha (Erα) expression in treated tissues compared to controls. Additionally, a decrease in apoptotic markers and an increase in cellular proliferation markers were observed. These findings suggest that exosome therapy can effectively enhance estrogen production and modulate receptor sensitivity in perimenopausal ovarian tissues. This approach could serve as a safer alternative to HRT, aligning with the body’s natural regulatory mechanisms and potentially offering a more effective treatment option for managing perimenopausal symptoms.

## 1. Introduction

The health challenge of navigating perimenopause represents a significant and complex issue affecting millions of women globally. As the precursor to menopause, perimenopause marks a transitional period that can span several years, during which women experience a wide range of symptoms that can profoundly impact their quality of life [1]. These symptoms not only include hot flashes, mood swings, and sleep disturbances but can also extend to weight gain, decreased libido, and cognitive changes such as memory lapses and difficulty concentrating [2]. The variability and intensity of these symptoms underscore the highly individualized nature of the perimenopausal experience.

Large-scale epidemiological studies have shown that nearly 80% of women report experiencing some form of perimenopausal symptoms. Additionally, the global burden of these symptoms is significant, with many women seeking medical help to manage their condition. For instance, data from the Study of Women’s Health Across the Nation (SWAN) highlighted the widespread prevalence and diversity of perimenopausal symptoms among different ethnic groups, further emphasizing the need for effective management [3].

The decline in estrogen levels during perimenopause is a central factor in the emergence of these symptoms [4]. Estrogen plays a critical role in numerous bodily functions, and its reduction can have systemic effects, laying the groundwork for an increased risk of developing chronic conditions such as cardiovascular disease, osteoporosis, and type 2 diabetes [5]. These long-term health risks add another layer of complexity to the management of perimenopause.

Currently, hormone replacement therapy (HRT) remains the most prevalent treatment for managing perimenopausal symptoms, offering relief by supplementing the body’s declining estrogen levels [6]. However, HRT’s applicability is not universal. Concerns over potential side effects, including an increased risk of breast cancer, blood clots, and stroke, particularly among certain groups of women, necessitate caution and limit its use [7]. This situation highlights a critical gap in the healthcare landscape—a need for innovative, safe, and personalized treatment options that can address the diverse and dynamic needs of women undergoing perimenopause. The quest for such treatments is not only about symptom management but also about enhancing overall well-being and preventing the onset of related chronic diseases, ensuring that women can navigate this natural transition with dignity and optimal health.

Exosomes have emerged as a promising diagnostic and therapeutic target for various reproductive diseases, including polycystic ovary syndrome (PCOS), primary ovarian insufficiency (POI), and several cancers such as breast and ovarian cancer [8,9,10]. Additionally, studies have demonstrated that exosomes can restore ovarian function in mice with non-obstructive azoospermia (NOA). These positive findings suggest that the transplantation of exosomes derived from mesenchymal stem cells (MSCs) holds significant potential as a treatment for combating ovarian aging [11].

Ovarian aging is closely related to menopause and perimenopause, primarily due to the critical role of estrogen in these processes. The decline in estrogen levels during perimenopause is a major factor contributing to the onset of menopausal symptoms and the associated health risks. By enhancing estrogen production and improving ovarian function, exosome therapy could offer a novel and effective approach to managing perimenopausal symptoms and mitigating the impacts of ovarian aging. We aim to pave the way for further research and advancements in this field, potentially providing new avenues for treating and understanding perimenopause and its related conditions.

## 2. Results

The genesis of our study was the procurement of ovarian tissue from 49-year-old females experiencing classic perimenopausal symptoms: irregular menstruation, hot flashes, and mood swings. Despite the lack of confirmatory follicle-stimulating hormone (FSH) levels, histopathological examination disclosed a diminished number or absence of follicles, pointing towards the underlying physiological disruptions of perimenopause.

**Figure 1:**
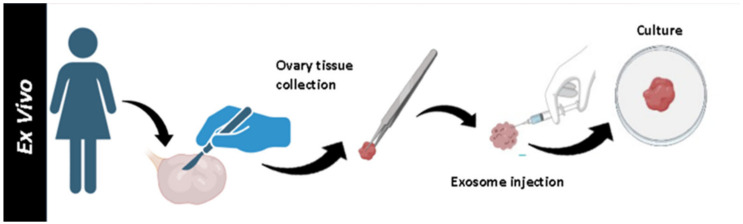
Representative phase images demonstrating the treatment of ovarian tissue to exosome ex-vivo.

Utilizing ex vivo methodologies, our research ventured into uncharted territories. Ovarian cortex specimens underwent treatment with INOFFA1 exosome (derived from human umbilical mesenchymal stem cells) injections (Figure 1).

**Figure 2:**
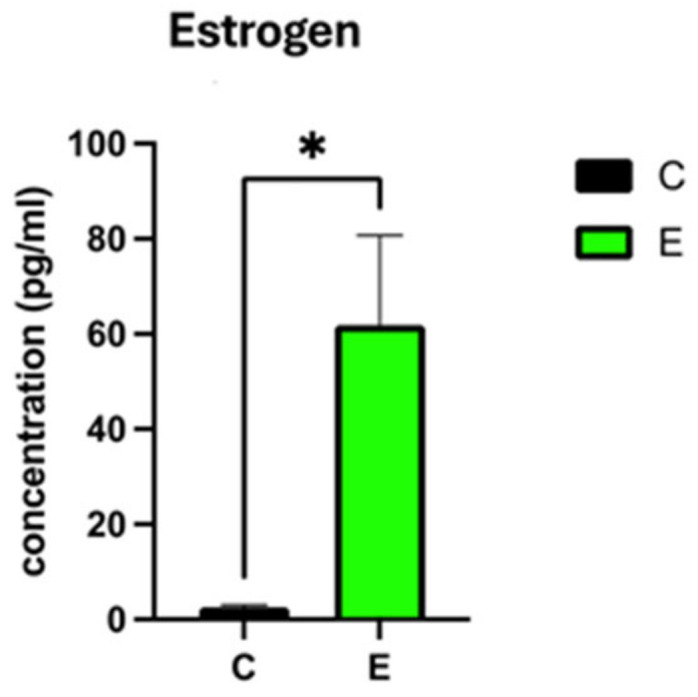
Estrogen level in the culture medium of ex-vivo exosome-treated ovary relative to the untreated control using ELISA technique. * indicate statistical significance according to student *t*-test (*p* < 0.05). The bar graphs represent the mean ± SEM of three technical replicate measurements of three independent biological replicates from three different patients.

Subsequent analysis revealed compelling evidence: a significant surge in estrogen levels in exosome-treated ovaries, as quantified by ELISA, marking a notable contrast to untreated controls. This elevation of estrogen (61.8 ±18.8 in treated versus 2.6 ± 0.41 in controls, *p* ≤ 0.05) underscores the potential of exosomes to mitigate the hormonal imbalance pivotal to perimenopausal symptomatology (Figure 2).

**Figure 3:**
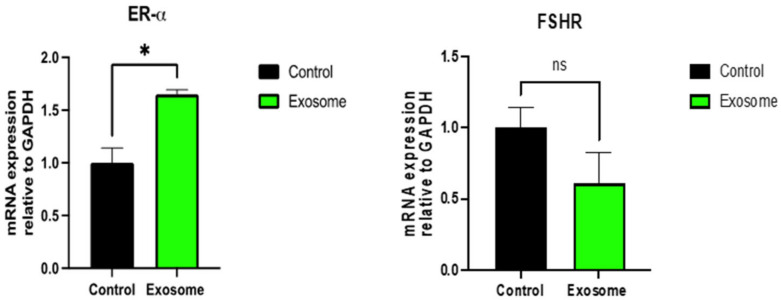
The relative changes in the gene expression of ER-alpha (**left**) and FSHR (**right**) in ex-vivo exosome-treated ovary relative to the untreated control were quantified by the 2−ΔΔCT method. * indicate statistical significance according to student *t*-test (*p* < 0.05); ns: nonsignificant. The bar graphs represent the mean ± SEM of three technical replicate measurements of three independent biological replicates from three different patients.

Further molecular analyses corroborated these findings. Quantitative PCR (q-PCR) illustrated a significant enhancement in estrogen receptor-alpha expression (1/2-fold increase), coupled with a marginal decrease (one-fold decrease) in FSH receptor levels in treated tissues (Figure 3). This suggests not only an increase in estrogen production but also a modulation of hormonal sensitivity at the tissue level.

**Figure 4:**
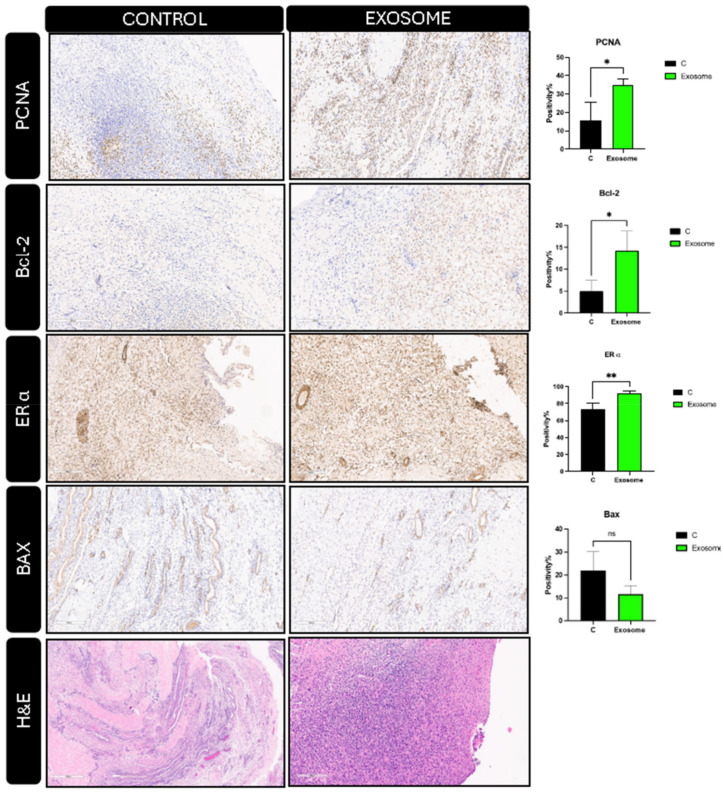
Histology and immunohistochemical staining of ex-vivo exosome-treated ovary relative to the untreated control; hematoxylin and eosin (H&E) staining (bottom); BAX, ER-a, Bcl-2, and PCNA (Top). The slides were scanned and analyzed using the Aperio ImageScope colocalization algorithm; Pathology Slide Viewing Software *, ** indicate statistical significance according to student *t*-test (*p* < 0.05, *p* < 0.01); ns: nonsignificant. The bar graphs represent the mean ± SEM of three technical replicate measurements of three independent biological replicates from three different patients.

Immunohistochemistry (IHC) provided additional insights, evidencing a significant uptick in markers indicative of cellular proliferation (PCNA) (19.25%) and anti-apoptotic activity (Bcl-2) (9.25%), alongside heightened Estrogen receptor-alpha expression (19%) (*p* ≤ 0.05, 0.05, 0.01), respectively in exosome-treated tissues. In addition, insignificant decrease in Bax as an apoptotic marker. (Figure 4). These molecular alterations herald a conducive environment for estrogen production and action, potentially counteracting the follicular paucity observed in perimenopausal ovaries.

## 3. Discussion

Recently, many researchers have noted MSC-derived exosomes due to their therapeutic potential as well as accessibility and wide availability [12,13,14]. Moreover, compared with the whole process of preparing and storing stem cells, exosomes offer an excellent feasible alternative as they are more accessible and less expensive to obtain [15,16]. In addition, the clinical trial using exosome-based therapy for reproductive disorders has already been approved. Most recently, our team successfully obtained FDA approval for the first IND study on exosome therapy for a reproductive disorder. The clinical trial is a first-in-human phase 1 pilot trial to examine the safety and efficacy of MSC-derived exosome therapy in patients with premature ovarian insufficiency. It is an open-label, prospective, interventional trial where single-dose exosome therapy will be administered intravenously to patients diagnosed with premature ovarian insufficiency (POI) based on the American Society of Reproductive Medicine guidelines, and enrollment is planned to start soon (IND 28896). For these reasons, among stem cell-based therapy options, exosome therapy is a more stable, safer, and cell-free approach that can be widely applied and studied in regenerative medicine clinical applications [17,18]. In addition, compared to stem cells, stem cell-derived exosomes possess numerous advantages, such as non-immunogenicity, non-infusion toxicity, easy access, effortless preservation, and freedom from tumorigenic potential and ethical issues [19]. 

Our project’s novel approach to addressing the challenges of perimenopause leverages the cutting-edge science of exosome therapy, utilizing these cell-derived vesicles as vehicles for enhancing the body’s natural estrogen production. Exosomes, essentially the body’s intercellular messengers, are equipped with the remarkable capability to facilitate communication between cells, playing a pivotal role in various physiological processes, including immunity enhancement, tissue regeneration, and the modulation of pain and inflammation. This capability positions them as ideal candidates for a targeted therapy aimed at addressing the core issues of perimenopause.

Derived from human umbilical mesenchymal stem cells, the exosomes we employ are specifically designed to target ovarian tissues, encouraging them to ramp up the production of estrogen naturally and our team has previous successful and ongoing trials exploring the treatment potential of exosomes in ovarian disorders [20]. This innovative strategy bypasses the need for external hormone supplementation, thereby potentially reducing the risks associated with traditional HRT. Our rigorous ex vivo research serves as the foundation for this approach, providing compelling evidence that targeted exosome therapy can significantly influence estrogen levels within the body.

By focusing on enhancing the body’s own estrogen production, this therapy promises a more natural, safer, and personalized way to manage perimenopausal symptoms. It represents a significant departure from the one-size-fits-all model of HRT, offering women a therapy that is tailored to their unique physiological makeup and needs. Moreover, this approach opens up new avenues for those who, due to personal or family health histories, are unable or unwilling to use traditional hormone therapies.

The implications of these findings are twofold. Firstly, they offer a glimpse into the underlying mechanisms through which exosome therapy enhances estrogen production and receptor sensitivity within ovarian tissues. Secondly, and more importantly, they pave the way for a novel, non-hormonal treatment modality for perimenopause. This approach not only circumvents the risks associated with traditional HRT but also aligns with the body’s natural regulatory mechanisms, offering a more harmonious solution to the challenges of this transition period [21,22,23].

Our study represents a significant advancement in understanding and addressing perimenopause. It provides preliminary baseline data for future clinical trials utilizing intravenous or intraovarian exosome injections in patients with perimenopause symptoms. Based on our scientific findings, we plan to initiate a larger-scale experiment with more tissue samples and apply this in mouse models to investigate the natural limitations and potential drawbacks.

By illuminating the potential of exosome therapy, we unlock new avenues for safer, more effective treatments, heralding a hopeful future for women’s health. The implications of these results are profound, suggesting a new horizon in the treatment of perimenopausal symptoms. By providing an alternative to HRT that is potentially devoid of its risks and side effects, exosome therapy could revolutionize the management of perimenopause, offering a safer and more effective treatment option. This study not only contributes to the growing body of evidence supporting the therapeutic potential of exosomes but also opens new avenues for research and development in the field of women’s health.

## 4. Materials and Methods

Our study employed an ex vivo approach, utilizing ovarian cortex specimens from three perimenopausal women, all aged 49, who had undergone hysterectomies (patients consented to the University of Chicago Tissue Bank Study (IRB20-1414)). We utilized mesenchymal stem cells derived from the human umbilical cord (hUC-MSCs) that were obtained from RoosterBio (Frederick, MD, USA). These cells were specifically isolated from the perivascular Wharton’s jelly region of the human umbilical cord. The cultivation and expansion of these cells adhered to the guidelines provided by RoosterBio and were conducted in RoosterNourish-MSC-XF medium (RoosterBio, Frederick, MD, USA) until the cells reached 80% confluence. After reaching the third passage at 80% confluence, the hUC-MSCs were washed three times with phosphate-buffered saline (PBS) and subsequently cultured with RoosterCollect-EV Pro™ medium (RoosterBio, Frederick, MD, USA) for 48 h. The resultant culture medium, enriched with secreted exosomes, underwent a series of centrifugation steps: 500× *g* for 5 min at 4 °C to eliminate cell debris, followed by 2000× *g* for 20 min to remove apoptotic bodies, and 10,000× *g* for 30 min toexclude microvesicles. The purification of exosomes was performed with the poly-ethyl glycol (PEG)-based precipitation method (ExoQuick-TC, System Biosciences, Palo Alto, CA, USA), which strictly adhered to the manufacturer’s protocol. Briefly, 1 mL of ExoQuick-TC was added to 5 mL of culture media, and the mixture was incubated overnight at 4 °C; ensuing centrifugation at 1500× *g* for 30 min resulted in the pelleting of exosomes. The resulting pellet was resuspended in 200 μL of PBS, and the quality and quantity of the exosomes were evaluated using NanoTracking Particle analysis with a NanoSight NS300 system (Malvern Panalytical, UK). The purified exosomes were stored at −80 °C and subjected to no more than one freeze–thaw cycle. To produce enhanced exosomes (INOFFA 1), we cultured hUC-MSCs with HGrC1 cells in accordance with a system protected by intellectual property rights (patent number: PCT/US2022/073467). The culture medium enriched with exosomes was harvested and subjected to a series of sequential centrifugation steps: centrifugation at 500× *g* for 5 min, 2000× *g* for 20 min, and 10,000× *g* for 30 min to eliminate debris, apoptotic bodies, and microvesicles, respectively. Exosome purification was carried out using ExoQuick-TC, strictly following the manufacturer’s instructions. The quality and quantity of the exosomes were thoroughly analyzed using a NanoSight NS300 system and characterized via Transmission Electron Microscopy (TEM) [20]. The exosomes used in this study were purified based on cGMP regulations, ensuring high safety standards. The ovarian tissue slices (2 mm × 2 mm × 2 mm), after placing on 200 μL Matrigel, were cultured in a medium consisting of advanced Dullbecco’s modified Eagle’s medium (DMEM/F-12) (Fisher Scientific, Waltham, MA, USA) [24] supplemented with 12% fetal bovine serum (FBS) and 1%peniciilin-streptomycin within 4 h after surgical resection. The specimens were treated with either 1.50 × 10^9^ particles/mL EV injections against phosphate buffer saline (PBS) as control and cultured at 37C, 5%CO_2_, and atmospheric oxygen level for 24 h.

After 24 h, the conditioned culture medium was collected and tested for estrogen level quantification using ELISA.

Part of the ovarian slices were fixed in 10% buffered formalin for at least 24 h at room temperature. Subsequently, tissue slices were embedded in paraffin, and 2 μm sections were generated and underwent IHC staining with PCNA (ab29), Bcl-2(ab59348), Estrogen receptor-alpha (ab3575), and Bax(Abcam, MA, USA).

The other part of ovarian slices undergoes RNA extraction using the miRNeasy Tissue/Cells Advanced Micro Kit Cat. No. 217684 (Qiagen, Valencia, CA, USA) was used according to the manufacturer’s instructions. Reverse transcription was conducted using the RNA to cDNA EcoDry™ Premix (Double Primed) Kit, Cat. No. 639549 (Takara Bio, CA, USA). RT-PCR was performed on a CFX Connect RT-PCR Detection System (Bio-Rad Laboratories, Hercules, CA, USA) using Advanced Universal SYBR^®^ Green qPCR Mastermix (TaKaRa, Tokyo, Japan).

The PCR primers FSHR and ER-alpha were used in the experiment, and the expression levels were normalized to those of GAPDH in each patient. The PCR amplification procedure included an initial pre denaturation step at 95 °C for 30 s, followed by 40 cycles of denaturation at 95 °C for 15 s, annealing at 60 °C for 30 s, and extension at 65 °C for 31 s. The relative expression of the target gene was calculated using the 2^−ΔΔCT^ method [25].

This methodological framework allowed for a comprehensive assessment of the potential for exosome therapy to enhance estrogen production in perimenopausal ovarian tissue.

## Data Availability

The raw data were generated at the University of Chicago. Derived data supporting the findings of this study are available from the corresponding author upon request. The data are not publicly available because they contain information that could compromise participants’ ethical approval and consent to participate.
